# Supplementary material for: Lived experience in mental health research in Ghana and Indonesia: What have we learned?
Source: PLOS Ment Health. 2025 Jun 17;2(6):e0000344. doi: 10.1371/journal.pmen.0000344 (PMC12798261; doi:10.1371/journal.pmen.0000344)
Supplement: Attachment — Submitted filename: Response to reviewers.docx [file pmen.0000344.s002.docx]

**Response to reviewers**

**PMEN-D-24-00484
Paper title: Lived experience in mental health research in Ghana and Indonesia: What have we learned?**

**Reviewer #1:** This is an interesting paper with thorough information, addressing various aspects. However, I believe it is too verbose, making it challenging to hold the reader's attention. It would be better to revise it before considering it for publication.

**Response:** Thank you for this observation. We agree that our paper is quite wordy and so we have cut some words and rephrased to be more succinct.

**Reviewer #2:** Overall this was an excellent exploration of the incorporation of those with lived experience into the research process especially as it pertains to mental health. I appreciated the plain language and practical considerations that were discussed within the paper. I saw the biggest strengths of the work to be the clarity of scope in terms of who exactly was included as peer researchers and the straightforward nature in which the difficulties of such an approach were described. The data used enriched my understanding of the research and humanized the research experience in a way that was apropos given the topic.

**Response:** Thank you for this feedback. Our approachable style reflects the varied academic backgrounds of the research team and we hope enables our paper to reach a wider audience.

I appreciated the recommendations section's (starting on line 723) categorization of the various needs when considering including lived experience within a research team, however, I felt that the section lacked enough specificity to be fully operationalized by researchers seeking to follow the suggestions.

**Response:** Thank you for this observation. We have added more detail in places, for example in relation to remuneration on pp40-41. However we feel that we cannot be too prescriptive in our recommendations as there will be variability dependent on the specific institutional and cultural context and the kind of research being conducted.

While the article is focused on the concept of including those with lived experience within mental health research the arts-based nature of the interventions is mentioned but not described in enough detail to fully understand what was done exactly. This may not be relevant given the focus of this particular article, but I thought it might have been helpful to more broadly understand what was asked of the peer researchers.

**Response:** Thank you for this helpful comment. In the methods and elsewhere in the paper we have clarified that research for the first project employed participatory video and provided examples of the participatory arts methods employed for the second project

In some sections of the article the "Global South" is discussed as potentially a unique environment in terms of challenges to implementation of lived experience inclusive research in mental health. However, I did not get enough information on why this may be the case nor did I get a sense as to whether the recommendations and discussion is valuable globally or more appropriate to this particular region.

**Response:** Thank you for this insight. We provide a summary of some of the additional challenges related to lived experience research in the Global South in the introduction and in the section on lived experience research in Ghana and Indonesia. We also describe where our team experienced particular difficulties related to Global North-South inequalities and point out where addressing these is relevant to our recommendations. Our argument is not that these challenges are necessarily unique to the Global South but are exacerbated due to the factors such as scarcity of resources, power imbalances resulting from colonial histories, weak infrastructure, difficulties in accessing funding and the novelty of lived experience involvement, with much fewer opportunities for paid employment in this field. We have added lines to this effect on pp38-39. We are aware of a number of published papers exploring lived experience engagement in research in the Global North, some of which we cite, but there are much fewer focusing on these additional challenges in the Global South.

I very much appreciated the acknowledgement that is made in the research team characteristics section (starting on line 220) that the diversity in terms of educational and socio-economic background was limited. I had hoped to see more discussion regarding the potential challenges of broad inclusion in mental health research of those with lived experience due to symptoms and challenges of the lived experience itself. The challenges section (starting on line 71) and in several other places in the paper seem to concentrate mostly on power systems not allowing participation, but I think that a more nuanced conversation would be helpful when considering that direct symptoms of mental health could be highly challenging to experience while engaging in the research process. Especially when attempting to research more severe mental health challenges, inclusion of those with lived experience in the design and implementation of the research is not just a hurdle of willingness and funding. This is briefly touched on in the section entitled democratize opportunities for participation, however, this section mostly indicates that it should be done without offering concrete solutions on how to do it.

**Response:** Thank you for raising this issue. Our argument is informed by a social model of disability where barriers are less to do with mental health conditions, than with the support and care provided. Our experience has been that with sufficient support and accommodations, people with serious mental health conditions are able to take an active and meaningful part in research. Of course, as with any serious health condition, there may be times when people are unable to work to their full capacity. We have touched on this issue in relation to a member of the team experiencing a mental health crisis during the research (p32) and some of us feeling triggered by the research process, particularly during participatory activities when they also needed to support others (p25). We describe the safeguarding procedures put in place to address these issues (p11) and the need to account for these support needs in our recommendations (p40). We avoided repeating these in the section on diversifying representation but have inserted a sentence at the end of this section (p44) to underline the responsibility to provide support for people even with profound difficulties to take part.

I very much enjoyed the article and am glad that this is being pushed more firmly into our consciousness as researchers in mental health. Thank you for your work and your insights. I hope that you are able to continue down this path and continue to do good scholarship that helps those that have been overlooked for far too long.

**Response:** Thank you for your encouragement – we are committed to continuing on this path and building on our research experience

**Reviewer #3**: Thank you for the opportunity to review this important article. The manuscript is well written and organized overall and very interesting findings that fit with meaningful inclusion of patients, public and people with lived experience of mental illness. This paper adds an important contribution to the literature in advancing PPI or Patient Oriented Research and addresses a large gap with inclusion of people with mental health conditions as peers in conducting research.

**Response:** Thank you for this encouraging feedback.

While the paper advances knowledge in this area, particularly from the Global South perspective, it is unclear how the thematic analysis was conducted? for example themes are described well with excerpts /examples but what kind of thematic analysis was conducted? e.g. Braun and Clarke reflexive thematic analysis? this might help add rigor to the methods section.

**Response:** Thank you for this suggestion. However we are not reporting on analysis of research data (for which we did indeed draw on Braun and Clarke’s approach to thematic analysis) – rather we are reporting on reflections and evaluation of the peer research process. As a consequence we did not follow a formal coding and analysis process. Braun and Clarke’s reflexive thematic analysis would not be appropriate as it involves the development of conceptual themes from data gathered from research participants, whereas we are reporting on subjective experiences as peer researchers. In order to address this and avoid confusion, we have removed the word ‘analysis’ and provided more detail on the process of developing the paper and the themes (p14).

On line 223 authors note "all peer researchers are graduates and 5 have master's degrees" it is unclear what graduates means? graduates of what? perhaps consider taking this out and simply saying 5 had graduate degrees?

**Response:** We have clarified this to state that almost all the peer researchers are university graduates (p15).

This adds more nuanced understanding of peer researchers as not only having lived experience of mental health conditions but also other experiences in which they can meaningfully contribute to research e.g. some may be engineers, lawyers, activists etc. See DOI:10.1186/s40900-021-00266-1

**Response:** Thank you for this very important point and for signposting us to this paper which we were not familiar with. We have added a sentence to recognise the varied skills within the peer research team which at various times contributed towards the research process, as well as their lived experience of mental health conditions (p16).

Finally, I appreciate the spotlight on low middle income countries and learned a lot about how people with mental illness are treated in the Global South. I find it interesting that many of the themes are relevant to the "Global North" and Global contexts. This may warrant policy directions related to thinking intersectionally about race, class, gender, geography etc. for policy on PPI in mental health research. A statement to this effect may strengthen the conclusions and overall findings and recommendations.

**Response:** We appreciate this point and have made some reference to thinking intersectionally about lived experience. We have added a sentence to strengthen this point (p44).

Overall, this manuscript and work is very informative,

Thank you for the opportunity to review the important work.

**Reviewer #4:** The abstract was well presented and summarized clearly explained what was done and the teams involved. It actually provided a clearer direction into the manuscript. However I felt there was need for the number of researchers to be mentioned in the abstract or in the early stages of the manuscript.

**Response:** Thank you. We realise this is unclear. We have added the numbers of peer researchers and academic researchers employed on both projects and writing the paper to the methods section (p10) as well as a table.

**Reviewer #5:** 1. General Assessment

The manuscript titled “Lived experience in mental health research in Ghana and Indonesia: What have we learned?” is a valuable contribution to the field of mental health research, particularly in low- and middle-income countries. It addresses the critical issue of involving lived experience researchers in a methodologically rigorous and ethically responsible manner. The paper aligns with the publication criteria of PLOS Mental Health and offers actionable insights for improving participatory research practices.

**Response:** Thank you for this positive feedback.

2. Technical and Methodological Soundness

The study employs participatory and arts-based methods to engage lived experience researchers in Ghana and Indonesia, effectively addressing an under-researched area in the Global South. The thematic analysis is conducted rigorously, with clear descriptions of how themes were identified and refined collaboratively between peer and academic researchers. The data collected support the conclusions, which are well-drawn and aligned with the study’s objectives. However, the authors should consider elaborating on specific challenges related to thematic saturation and intercultural translation issues to further strengthen the methodological discussion.

**Response:** Thank you very much for this suggestion. However we are not reporting on analysis of research data – rather we are reporting on our subjective reflections and evaluation of the peer research process. As a consequence we did not follow a formal coding and analysis process. Thematic saturation would therefore not be an appropriate approach and is not supported by some experts in thematic analysis such as Braun and Clarke as it reflects a positivist orientation, rather than an interpretivist approach (i.e. there is always another way of interpreting the data). To avoid confusion, we have removed the word ‘analysis’ and provided more detail on the process of developing the paper and the themes (p14).

Written reflections by both teams were in English. As we describe, some verbal discussions within the Indonesian team were in Indonesian but inter-country discussions were in English. All the peer researchers had some fluency in English and Indonesian researchers translated their reflections from Indonesian to English where needed. We have added this information to the methods section (p15).

3. Statistical Analysis

This is a qualitative study and does not include statistical analysis. The qualitative methods used are appropriate and rigorously applied. The authors’ triangulation of multiple data sources, such as feedback forms, reflections, and discussions, ensures the robustness of the findings.

4. Presentation and Clarity

The manuscript is well-written and presented in standard English. It is clear, concise, and accessible to a broad audience, including readers who may not be familiar with participatory research. The structure—comprising abstract, introduction, methods, findings, discussion, and recommendations—is logical and easy to follow. That said, some sections, particularly the discussion, could benefit from additional specificity about the applicability of the findings beyond the immediate contexts of Ghana and Indonesia.

**Response:** We have made some edits to emphasise where we believe our findings are relevant, particularly to peer research in the Global South. Our argument is not that these challenges are necessarily unique to the Global South but are exacerbated due to the factors such as scarcity of resources, power imbalances resulting from colonial histories, weak infrastructure, difficulties in accessing funding and the novelty of lived experience involvement with much fewer opportunities for paid employment in this field. We have added lines to this effect on pages 38-39. We are aware of a number of published papers exploring lived experience engagement in research in the Global North, some of which we cite, but there are much fewer focusing on these additional challenges in the Global South.

5. Ethical Considerations

The manuscript demonstrates strong adherence to ethical standards. It includes detailed information on ethical approvals obtained and the informed consent process. The authors also protect participant anonymity by using identifiers rather than names. The manuscript emphasizes the need for equitable remuneration and support for peer researchers, addressing potential concerns about exploitation or tokenism.

6. Strengths

The focus on lived experience researchers in the Global South is novel and addresses a significant gap in the literature.

The participatory methods employed enrich the research process and outputs, offering valuable lessons for future studies.

The recommendations are practical and grounded in the lived experiences of the participants, making them actionable for researchers and policymakers.

7. Areas for Improvement

Capacity Building: The manuscript mentions the importance of training for peer researchers but could provide more detail about the gaps in the training provided and specific ways future studies can address these issues.

**Response:** Thank you for this helpful suggestion. Peer and academic researchers identified various training needs which are discussed on pp29-30 and 33-34. We have added a sentence on p37 and p41 to summarise training needs for peer researchers to participate equitably at all stages of the research process. We have also inserted a sentence to recognise that capacity building should be provided for academic researchers to work meaningfully, ethically and effectively with peer researchers (p42).

Budgetary Considerations: While the authors highlight financial challenges faced by peer researchers, including delayed payments, they could provide more concrete suggestions on how funding mechanisms can better accommodate the needs of peer researchers.

**Response:** We have provided some more detail in the paragraph on fair and comprehensive remuneration on pp40-41.

Limitations: The authors acknowledge some limitations, but the discussion could benefit from a deeper exploration of how intersectional factors, such as language barriers and socio-economic status, might have influenced the findings.

**Response:** Thank you for this insightful feedback. We have added a sentence to highlight some of these intersectional factors (p44) and refer to the disadvantage faced by the Indonesian team in terms of the use of English (p16). However due to space limitations and to avoid repetition, deeper discussion of issues around power inequities are discussed in the findings and inform the recommendations.

8. Recommendations for Publication

I recommend the manuscript for publication with minor revisions to strengthen the discussion and provide additional detail in the methods and recommendations sections.

9. Final Comments

This paper makes an important contribution to the field of mental health research, particularly in ensuring more equitable and inclusive practices in the Global South. The findings are timely and relevant, offering valuable guidance for researchers, practitioners, and policymakers. With the suggested revisions, this paper has the potential to significantly influence future research practices.

**Reviewer #6:**

Overall: This manuscript addresses an important and under-captured topic. The authors share meaningful insights and practical recommendations for peer research. In particular, the authors provide tangible lessons and recommendations that may be useful to others wanting to integrate participation but not knowing how to do so safely. However, certain aspects of the manuscript could be strengthened to enhance clarity, coherence, and impact. The structure and framing of the results could be improved; reducing repetition and creating clearer links between sections would enhance readability and ensure that the core messages are not lost.

**Response:** Thank you for this helpful suggestion. We have revised the manuscript for greater clarity and removed some of the repetition.

The manuscript currently presents findings as though peer researchers were participants rather than co-authors. I encourage the authors to reconsider the framing of the results to reflect the collaborative development of the manuscript.

**Response:** We are grateful for this important insight and we have reframed parts of the paper to reflect this. However we found this challenging as we are writing as a team of researchers, some with and some without lived experience of mental health conditions. Therefore we could not write entirely in the first person without misrepresenting our different roles and experience. We acknowledge the difficulties this presents and we hope to address this in future publications as some of us with less experience gain more skills in leading academic writing.

Additionally, expand on how the team defines "peer researcher" providing detail on the extent of involvement, roles, and responsibilities, particularly, as these appear to vary across peer researchers.

**Response:** We describe the roles and responsibilities of the peer researchers in the methods under ‘peer researcher activities’. We have clarified the differences in these roles/activities across the projects/settings and provided a table.

Overall, I appreciate the author’s thoughtfulness, careful considerations and look forward to seeing the revised manuscript.

**Specific Comments**

Abstract (pg. 3): The sentence beginning, "Based on these experiences..." could benefit from re-wording for clarity. Consider breaking it into shorter sentences or rephrasing for greater readability.

**Response:** Thank you for this feedback – we have reworded this sentence and separated it into two.

Introduction (line 30): The terms "Patient and Public Involvement (PPI), community engagement, co-production, participatory research, and peer research" represent distinct approaches and dynamics. Consider briefly clarifying their differences or focusing on the most relevant terms for this study.

**Response:** We agree and have removed this sentence to focus on defining peer research which is the topic of our paper.

Introduction (lines 62-65): While lived experience may enhance empathy and understanding, this assertion would be stronger if supported by evidence – as lived experience is not a necessary precursor for empathy and understanding. Clarify whether there is an assumption of increased "reliability and authenticity", is this based on the assumption or expectation that there will be disclosure of lived experiences? Reflect on the potential risks to emotional safety for peer researchers (and participants) if disclosure becomes an implicit expectation.

**Response:** We appreciate this point and have rephrased this section. Later in the paper we point out that peer researchers and participants with mental health conditions may have very divergent experiences (p34, pp43-44). We also acknowledge the potential risks of expectations for peer researchers to disclose their experience. We discuss this in some detail under challenges and recommendations but have added a sentence to the introduction under challenges (p7) to highlight this point.

Introduction: Consider including mention of the importance of participation and the risks of extractive processes in collaboration in contexts where there is a risk of extractive processes, particularly when Global North institutions benefit from working in Global South contexts.

**Response:** Thank you for this insightful comment. We have added a sentence to this effect on p6, noting that peer researchers may be particularly vulnerable to these extractive processes.

Methods (line 158): For the WHO Recovery Planning template, as this is part of a self-help toolkit, clarify whether this was for personal reflection (e.g. as a personal safety plan) or if peer researchers were expected to share this information.

**Response:** This information was shared with the research team as part of safeguarding so that we could support peer researchers to access help according to their stated preferences if needed. We have added this information on p11.

Methods (line 178): Provide additional details on the extent of collaboration among peer researchers, academic researchers, and artists in the second project. Specify what is meant by "involved."

**Response:** We have rephrased this section and added more detail on the role of the peer researchers in this project (pp12-13). This is also included in the table.

Methods (line 198): Expand on how the themes were chosen, detailing decision-making processes and how balance and integration of perspectives (e.g. the integration of Indonesian peer researchers experience into the draft developed by the Ghana team)?

**Response:** We have revised this section to provide some more detail of the process of developing the paper and themes (p14)

Results: At times the wording shifts between insider and outsider (e.g. line 308 “This confidence was evidenced during participatory thematic analysis of interviews for the first project when peer researchers engaged in lively debates with academic researchers.”) Consider the wording of the results to reflect the collaborative nature of the development of the manuscript (rather than peer researchers as participants).

**Response:** We are grateful for this important insight and we have reframed and reworded parts of the paper to address this issue. However we found this challenging as we are writing as a team of researchers, some with and some without lived experience of mental health conditions. Therefore we could not write entirely in the first person without misrepresenting our different roles and experience. We acknowledge the difficulties this presents, particularly in creating confusion between peer researchers and research participants.

Results (line 412 “These were not openly discussed by all the peer researchers but were also observed by the academic researchers in their own reflections on the process.”): Expand on whether the shared challenges were collaboratively agreed upon by all researchers. Consider distinguishing challenges experienced by peer researchers from those perceived or experienced by academic researchers.

**Response:** We have expanded on the process of developing the themes in the methods (p14). We have also edited the findings to distinguish where challenges were identified by peer researchers in Indonesia and/or Ghana or by the academic researchers.

Results (line 419): Clarify what is meant by “supporting others”. Peer researchers are not peer support workers; explore perceptions or expectations of their roles, such as caregiving or participant support (e.g. line 427 “I needed to be a caregiver”)

**Response:** Thank you for this insightful reflection. At times the role of peer researcher did overlap with peer support as members of the participatory groups for the second project could require mental health support. While the academic researchers who are also trained as psychologists provided this support during the group meetings, outside of the meetings, for example in WhatsApp groups, peer researchers could end up providing informal support and reassurance. This is a particular issue where participatory group members may not easily be able to access professional psychological support. In Indonesia peer researchers were also engaged in quite an intense period of preparation for the performance. This is something that we acknowledge should have been considered in more detail in planning for the second project, for example providing training in mental health first aid or peer support (p34). We have edited this section to clarify what is meant by ‘support’ within the context of the participatory group meetings (pp25-26) and provided more detail on the additional roles for the peer researchers in Indonesia (p13).

Results (line 580 “academic researchers were also experienced psychologists”) Provide additional context - was this part of a pre-planned safety plan? How were dual roles managed and safe boundaries around providing psychological support maintained?

**Response:** We appreciate this comment. In developing the research proposals to include peer researchers the academic research team felt that the experience of the academic team as trained psychologists was a benefit in relation to safeguarding, particularly in contexts where professional mental health support can be hard to access. Peer researchers in Ghana commented on this. In structuring the research teams and meetings, open dialogue around boundaries and safeguarding preferences were frequently revisited. While the academic researchers/psychologists provided support in terms of facilitating emotional processing and reflection during daily debriefing sessions, these were not conceptualised as replacements for the peer researchers’ existing service use. When needed, peer researchers were supported to access professional services outside of the study context. The operational protocols that were developed at the beginning of the studies included discussions of this process. We have added a sentence on how the dual roles and responsibilities as psychologists/researchers were managed (p11).

Discussion (line 754): Consider discussing how differences in renumeration might perpetuate power imbalances or be perceived as valuing some opinions over others.

**Response:** We have added a sentence to clarify that differences in salaries for peer researchers would be related to their skills and experience, reflecting that they bring not only their lived experience of mental health conditions, but other skills and work experience (p41).
